# Exotic Megaherbivores as Ecosystem Engineers in Australian Savannas: Do They Facilitate Predator Movement?

**DOI:** 10.1002/ece3.71622

**Published:** 2025-07-09

**Authors:** Georgina Neave, Brett P. Murphy, Tiwi Rangers, Hugh F. Davies

**Affiliations:** ^1^ Research Institute for the Environment and Livelihoods Charles Darwin University Casuarina Northern Territory Australia; ^2^ Tiwi Resources Pty Ltd Casuarina Northern Territory Australia; ^3^ School of Environmental and Rural Science University of New England Armidale New South Wales Australia

**Keywords:** dingo *(Canis familiaris)*, feral cat *(Felis catus)*, *game trails*, megaherbivores, predator movement

## Abstract

An understanding of how terrestrial mammalian predators use their environment is critical for the development of effective management and monitoring. Mammalian predators often use anthropogenic linear features—such as roads, fencelines, and infrastructure corridors—to increase movement efficiency and prey encounter rates. However, there has been little investigation into how predators use more subtle linear features such as game trails (i.e., well‐trodden paths created by megaherbivores). This is despite native and exotic megaherbivores being abundant across many of Earth's most intact landscapes and conservation areas. We investigated how the two largest terrestrial mammalian predators in northern Australian savannas—the dingo (*Canis familiaris,* introduced ca. 4000 years ago) and cat (*Felis catus,* introduced ca. 200 years ago)—use game trails created by exotic megaherbivores (Asian water buffalo *Bubalus bubalis* and horse *Equus caballus* ). We deployed two camera traps at 52 sites, with one camera positioned on a game trail and another in undisturbed vegetation < 60 m away. We compared the activity of predators on game trails to adjacent undisturbed vegetation and explored how trail use varied with vegetation structure and prey activity. Dingoes and cats were 34 times and 6 times more likely to be detected on game trails than in adjacent vegetation, respectively, suggesting these predators preferentially use game trails. We speculate that the extensive network of game trails created by exotic megaherbivores across northern Australia's vast savannas has potentially facilitated terrestrial mammalian predator movement at very large scales. Controlling exotic megaherbivores may, therefore, provide a means of disrupting the activity of dingoes and cats, thereby benefiting predation‐susceptible native species. However, further research is needed to understand the ecological implications of game trails in Australian savannas and other habitat types.

## Graphical abstract

We investigated how two terrestrial mammalian predators in northern Australian savannas—the dingo ( *Canis familiaris* ) and feral cat ( *Felis catus* )—use game trails created by exotic megaherbivores. Using camera traps deployed at 52 sites, we found that both dingoes and cats were significantly more likely to be detected on game trails than in adjacent undisturbed vegetation, suggesting a preference for using the trails. We speculate that these trails may function as micro‐corridors that enhance predator movement and potentially increase their impacts on native fauna. However, further research is needed to fully understand the ecological implications of this association.

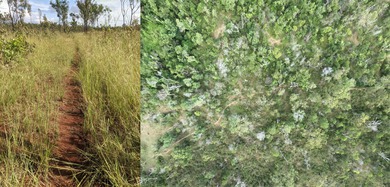

## 1 Introduction

Predators play a critical role in shaping terrestrial ecosystems, with their ecological function influenced by factors such as prey availability, interspecific interactions, and habitat structure (Pigeon et al. 2020). Increasingly, anthropogenic pressures—such as direct persecution and habitat modification—have altered predator behavior, movement patterns, and spatial dynamics, with consequences for native prey (Beschta and Ripple 2009; Ripple et al. 2014). One key way in which habitat structure can affect predators is through the availability of linear features that facilitate movement and foraging.

Terrestrial predators have been shown to preferentially use anthropogenic linear features including roads, railways, and infrastructure corridors such as powerlines and pipelines (Abrahms et al. 2016; Bischof et al. 2019; Dickie et al. 2017; Finnegan et al. 2018). Linear features can have a strong influence on predator movement with subsequent effects on predator–prey interactions (DeMars and Boutin 2018). For example, in the forests of North America, linear features such as seismic testing lines, pipelines, and roads associated with forestry and mining activities are extensive (Pasher et al. 2013). In this system, grey wolves ( *Canis lupus* ) preferentially use linear features (Finnegan et al. 2018; James and Stuart‐Smith 2000; Latham et al. 2011), along which they move up to 2–3 times more efficiently compared to through natural forest (Dickie et al. 2017). This efficiency enables wolves to increase their search rate and daily distances travelled (Dickie et al. 2020), increasing prey encounter rates (Latham et al. 2011; McKenzie et al. 2012; Whittington et al. 2011), with likely subsequent population‐level impacts on a threatened prey species, the woodland caribou (*Rangifer tarandus caribou*) (James and Stuart‐Smith 2000). A predator's preference for linear features is often thought to reflect optimal foraging theory, whereby the predator will adjust its behaviour to maximize energy gain and/or minimize competition and mortality risk (MacArthur and Pianka 1966; Pyke et al. 1977).

Global research investigating the influence of linear features on terrestrial predators has primarily focused on conspicuous anthropogenic features, and largely overlooked more subtle linear features, including those created by other fauna species. One such example is “game trails”—the well‐trodden paths created by large mammalian herbivores (henceforth referred to as megaherbivores). Megaherbivores are abundant in many of Earth's landscapes, including both highly modified and largely natural landscapes, where they often create dense networks of well‐defined game trails. We hypothesise that game trails can act in the same way as conspicuous anthropogenic features, such as roads and tracks, and facilitate the movement of predators throughout the landscape, and potentially even help to sustain predator populations. If this were true, it could be an important, but largely overlooked mechanism by which megaherbivores act as “ecosystem engineers”—organisms that create, modify, maintain, or destroy habitat conditions and resource availability, thereby significantly influencing the structure and function of an ecosystem (Briones 2024; Jones et al. 1994). While the loss of ecosystem engineers can lead to far‐reaching ecological cascades and biodiversity loss (Albertson et al. 2024; Davidson et al. 2012), the introduction of exotic ecosystem engineers could have similar effects in disrupting native community structure (Crooks 2002; Kamaru et al. 2024; Rilov et al. 2024).

Australia provides an excellent model system to study ecological cascades caused by the introduction of species by humans. Humans arrived 47,000–65,000 years ago (Clarkson et al. 2017), and the demise of the continent's functionally diverse megafauna followed (Gillespie et al. 2006). By ca. 45,000 years ago, no terrestrial animals with > 45 kg body mass remained (Roberts et al. 2001). Beginning ca. 4000 years ago, with the introduction of Australia's “native dog”, the dingo ( *Canis familiaris* ), a number of ecological niches have been filled by exotic mammals (Bowman et al. 2010). The British, who colonised the continent in 1788, introduced mesopredators, including the domestic cat ( *Felis catus* , hereafter referred to as feral cat) and red fox ( *Vulpes vulpes* ) (Abbott et al. 2014), along with a variety of megaherbivores. Both of these groups are now abundant across the entire continent. The combined effects of predation and habitat modification by exotic megaherbivores have led to remarkably high rates of extinction of Australia's native terrestrial mammals, with ~10% of mammals present in 1788 now extinct (Woinarski et al. 2015; Woinarski, Braby, et al. 2019).

Managed and unmanaged/wild exotic megaherbivores, such as cattle (*Bos* spp.), horses ( *Equus caballus* ), donkeys ( *Equus asinus* ) and buffaloes ( *Bubalus bubalis* ) are widespread and abundant across northern Australia's vast savanna landscapes (1.9 million km^2^) (Freeland 1990). Due to their large body size (> 300 kg) and hard hooves, the repetitive movement of these megaherbivores creates and maintains a network of well‐trodden paths through the grass‐dominated ground layer. While exotic megaherbivores have been implicated in the decline of native mammals across northern Australia (Legge et al. 2011; Woinarski et al. 2011), their contribution has primarily been attributed to broad‐scale habitat modification and degradation (Mihailou and Massaro 2021), as well as increased predation pressure resulting from the prolonged post‐fire suppression of ground‐layer vegetation due to grazing (Legge et al. 2019; McGregor et al. 2014). The abundance of exotic megaherbivores in Australia's savannas means that the potential network of game trails is substantial, even in the most remote areas where anthropogenic linear features are sparse. It is important to note that game trails are not unique to areas containing exotic megaherbivores; native macropod species can also create trails through habitual use. However, these trails are typically narrower, less persistent, and less spatially extensive than those formed by larger, exotic ungulates. While cats, dingoes, and foxes have been shown to preferentially use anthropogenic linear features across various Australian habitats (Dawson et al. 2018; Geyle et al. 2020; Raiter et al. 2018), their use of game trails remains largely unknown.

Here, we focus on a trophically simple model system, Melville Island, within Australia's monsoon tropics, to explore the hypothesis that megaherbivores can facilitate the movement of predators via the creation of game trails. We compared predator activity on game trails with that in adjacent vegetation, and hypothesized that predators would be most active on game trails, especially through areas of dense vegetation, while small native mammals would tend to avoid game trails. Although it is trophically simple, Melville Island is of high conservation significance, harboring important populations of many threatened species, especially mammals, that have declined significantly across the mainland. Understanding interactions between the island's native and exotic mammal species is critical to maintaining its biodiversity values.

## 2 Materials and Methods

### 2.1 Study Area

**FIGURE 1:**
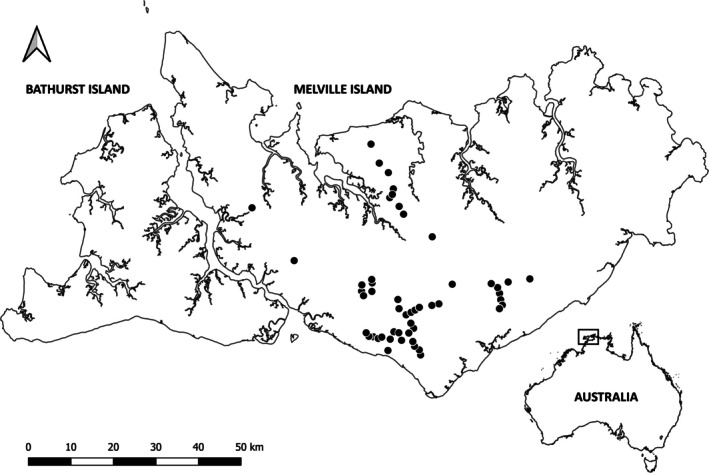
Location of the 52 sites (black circles) surveyed in 2022 on Melville Island. The location of Melville Island, relative to mainland Australia is indicated.

The Tiwi Islands are situated 60 km north of the city of Darwin in northern Australia (Figure 1). Melville Island—Australia's second largest island (5786 km^2^)—is situated in the wet–dry monsoon tropics and receives mean annual rainfall of 1400–2000 mm. The island is largely ecologically intact, with approximately 2% of native vegetation cleared for two small townships and other infrastructure, and 4% cleared for forestry plantations. The native vegetation is primarily dense savanna, dominated by eucalypts (*Eucalyptus* and *Corymbia* spp.). Understorey vegetation typically contains perennial C_4_ grasses, with variable shrub cover depending on the rainfall and fire history. Fire frequency is extremely high, with an average of 41% of the island burning each year.

Over the past two centuries, a range of exotic mammal species have been introduced to Melville Island, including water buffalo (ca. 7500 individuals; G. Neave, aerial survey unpublished data), horse (ca. 575 individuals; G. Neave, aerial survey unpublished data) and pigs ( *Sus scrofa* ) which are confined to the western half of Melville Island. Feral cats are widespread and occur in densities similar to mainland northern Australia (0.10–0.21 cats km^−2^, Davies et al. 2021). Dingoes are widespread on Melville Island (Neave et al. 2024) and in this context, are considered a native predator with cultural importance for Tiwi people. There has been no broadscale persecution of dingoes on the Tiwi Islands.

### 2.2 Data Collection

**FIGURE 2:**
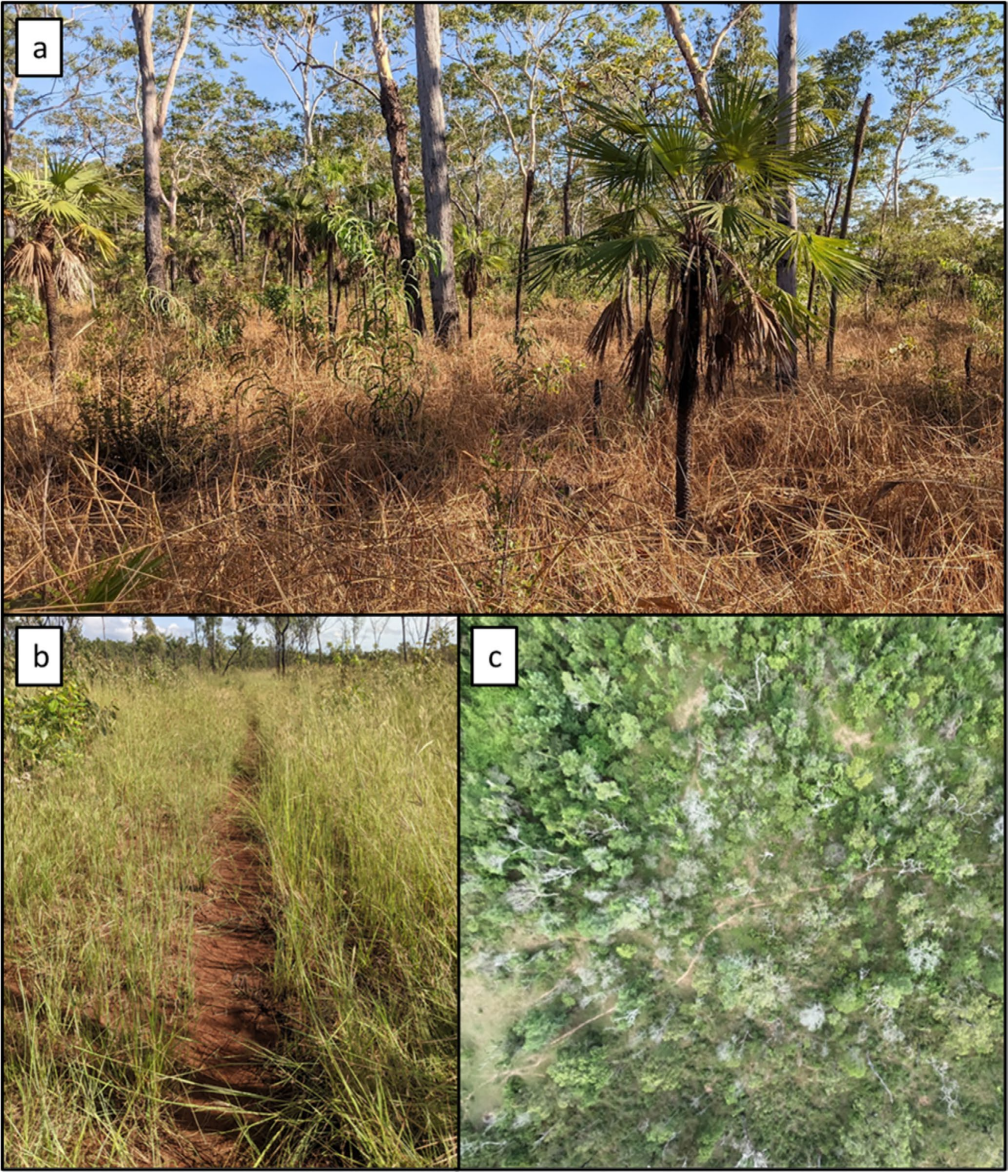
Open‐forest savanna vegetation on Melville Island (a), a game trail used in this study (b), an aerial photo of a game trail on Melville Island (c).

In 2022, we deployed two motion‐activated camera‐traps at 52 sites (*n* = 104 cameras) on Melville Island in eucalypt open‐forest or woodland (both a form of the savanna biome, with open canopy and grassy understorey) (Figure 1). Cameras were deployed in April, when vegetation biomass is highest, game trails are most pronounced (Figure 2), and fire scars are generally absent. Each site was > 1 km apart to minimise the likelihood of repeated detections of the same individuals. At each site, one camera was placed facing diagonally across a game trail and one camera in undisturbed vegetation < 60 m away, but not within 60 m of another adjacent game trail. Both cameras were unbaited and vegetation within the field of view was cleared to minimise false triggers. Cameras were faced horizontally, with “on‐trail” cameras directed at the centre of the game trail, while “off‐trail” cameras (hereafter referred to as on‐trail/off‐trail) were directed at the centre of the cleared area approximately 2 m from the camera. Two infrared camera model types were used (Reconyx Hyperfire PC800 and Reconyx Hyperfire 2 Pro Covert; Reconyx, WI, USA) across the study area, with the same model type used within a site for the paired on‐trail and off‐trail cameras. Each camera was set at 50 cm above the ground on a tree or metal post and programmed to take five consecutive images per trigger, with no quiet period between triggers, and sensitivity set to high. Cameras were deployed for a minimum of 36 days (range: 36–86 days) with both cameras at each site deployed and retrieved on the same day. Our study design closely follows that of Dawson et al. (2018), but with a key improvement: camera sites in our study were spaced 1 km apart, compared to 50 m in the original design. This greater spacing enhances the spatial independence of detections by reducing the likelihood that the same individual is recorded at multiple sites within a short timeframe.

Game trails selected for inclusion in this study were clearly defined pathways showing visible signs of recent use by exotic megaherbivores, such as fresh dung and track imprints with sharply defined edges. Trails were > 30 cm wide and extended for a minimum distance of 150 m (Figure 2). We developed a vegetation index to account for differences in ground layer vegetation density between sites. At each site, a 50 m transect was established along the game trail, extending 25 m to either side of the camera trap. Every 5 m along the transect, an observer crouched down to achieve an eye‐level height of 50 cm from the ground and looked towards a pole positioned 5 m perpendicular to the trail. The observer recorded the height at which the pole was completely obscured by vegetation. These measurements were then averaged for each site.

### 2.3 Image Processing

Camera images were first processed in MegaDetector (MDv5a, Microsoft 2023) to classify each image as being empty or containing an animal (Beery et al. 2019). For images classified as containing an animal, we used Timelapse image processing software (Greenberg et al. 2019) to tag images to species level. When small mammals (< 250 g body mass) could not be confidently identified to species, they were identified to genus. This was the case for *Sminthopsis* spp., *Pseudomys* spp., and *Rattus* spp.

### 2.4 Data Analysis

A large portion (33%) of our survey sites were burnt before the end of the sampling period. To limit the possible confounding effects of fire on game trail use, we only included data that were recorded before a site was burnt. To ensure even survey effort between paired cameras within a site, if one camera failed, then the earliest failure date was used as the survey end for both paired cameras. As one on‐trail camera malfunctioned after 2 days, this site was excluded, resulting in a total of 51 sites included in our analyses.

To estimate species activity, we calculated the number of independent detections of eight different taxa on the off‐trail and on‐trail cameras at each site: cat, dingo, buffalo, horse, black‐footed tree‐rat (*Mesembriomys gouldii melvillensis*), northern brown bandicoot ( *Isoodon macrourus* ), northern brushtail possum (*Trichosurus vulpecula arnhemensis*) and agile wallaby (*Notamacropus agilis*) and one grouping of taxa termed ‘small mammals’ (included all records of *Sminthopsis* spp., *Pseudomys* spp. and *Rattus* spp.). Detections were considered independent when > 30 min had elapsed since the previous detection of that species on the same camera. For sites where a species was detected, we compared activity by calculating the mean number of independent detections on on‐trail and off‐trail cameras over the full camera deployment period.

We investigated each species' use of game trails using logistic generalized linear models. The modeled response variable was the proportion of independent on‐trail detections relative to all independent detections recorded at each site. Sites with no detections of dingoes or cats on either camera were removed from the analysis. Models were fit with a quasibinomial error structure using the VGAM package (Yee 2015) in R (version 4.3.1, R Core Team 2023) to account for overdispersion. To examine how biotic variables influenced a species' use of game trails, we analyzed all combinations of the explanatory variables outlined in Table S1, with no model interactions as none were deemed ecologically relevant. Explanatory variables were checked for collinearity and standardized prior to analysis (Zuur et al. 2010). We performed model selection using the Quasi‐Akaike Information Criterion (QAIC), rather than the Akaike Information Criterion (AIC) because there was evidence that the models were over‐dispersed. Where no single model was clearly superior (i.e., multiple models with a ΔQAIC of ≥ 2 units), we used the model with the lowest QAIC to predict the proportion of detections on game trails.

## 3 Results

There were 4557 independent detections of the study taxa over 6298 camera trap nights. The most frequently detected species was the agile wallaby (2045 detections) and the most widely detected species was buffalo (98% of sites) (Table S2). Dingoes were recorded at 39 of 51 sites (76%), with 386 total detections. Feral cats were recorded at 26 of the 51 sites (51%), with 82 total detections. Northern brushtail possum and northern brown bandicoot were the most commonly detected small to medium‐sized native mammals, with 940 independent detections across 82% of sites and 332 detections across 69% of sites, respectively. Smaller native mammals from the genera *Pseudomys*, *Rattus* and *Sminthopsis* were infrequently detected (a total of 25 detections) and were only used in analyses when combined as one explanatory variable: “native mammalian prey” (Table S1).

**FIGURE 3:**
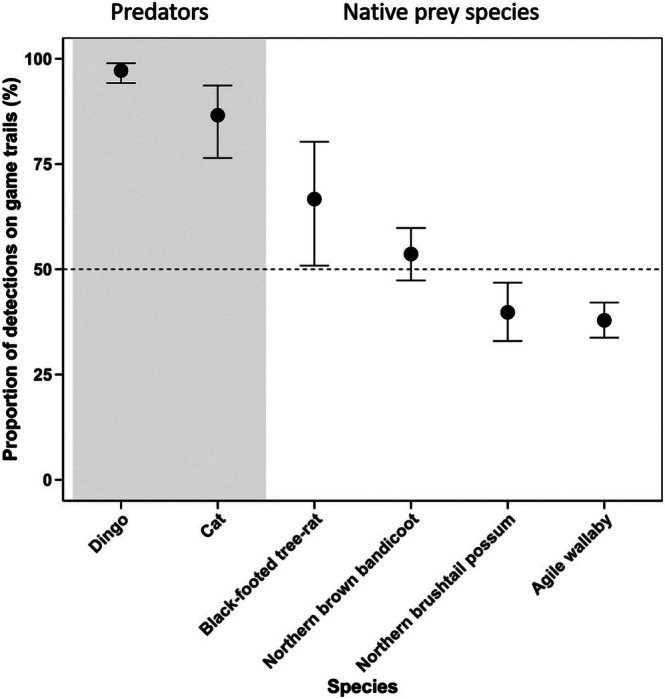
The proportion of total camera detections on game trails for each species. Fitted values with 95% confidence intervals. Values above the horizontal dashed line indicate a preference for game trails, while values below suggest an avoidance of game trails. Species are grouped into predators (grey shading) and native prey species.

Dingoes had the highest proportion of detections on game trails across all taxa, with 97% of detections occurring on trails (Figure 3). The mean number of detections on game trails over the full camera deployment period was 9.6 (±2.7 SE), compared to 0.3 (±0.1 SE) in adjacent vegetation. Feral cats were also more frequently detected on game trails, with 86% of detections on trails (Figure 3). The mean number of detections over the full deployment period on game trails was 2.7 (±0.6 SE), compared to 0.4 (±0.1 SE) in adjacent vegetation.

Native mammals, the northern brushtail possum and agile wallaby were more frequently detected on off‐trail cameras, with 60% and 62% of detections on off‐trail cameras, respectively (Figure 3). The northern brown bandicoot had similar detection rates on game trails and in adjacent vegetation, with 53% of detections on game trail cameras (Figure 3). The black‐footed tree‐rat had a higher proportion of detections on game trails, with 66% of detections on trails (Figure 3). While no clear correlates of game trail use were identified for seven of the eight study taxa (Table S3), the proportion of northern brushtail possum detections on trails was positively correlated with vegetation density.

Exotic megaherbivores exhibited high rates of detection on trails (90% and 81% of detections were on trails for buffalo and horse, respectively), and this is consistent with game trails being created by megaherbivores. The mean number of buffalo detections per camera over the full deployment period was 8.6 (±1.1 SE) on game trails, compared to 1.0 (±0.2 SE) in adjacent vegetation. The mean number of horse detections on and off game trails was 4.8 (±0.9 SE) and 1.2 (±0.3 SE), respectively. The next largest animal, the agile wallaby, had a higher proportion of detections on off‐trail cameras.

## 4 Discussion

An extensive body of literature has demonstrated that some terrestrial predators preferentially use linear features (James and Stuart‐Smith 2000; Latham et al. 2011; Dickie et al. 2020) However, most studies have focused on prominent anthropogenic features such as roads, tracks, and seismic testing lines, with little attention given to subtle linear features, such as the network of game trails created by exotic megaherbivores. We hypothesised that in northern Australian savannas, game trails could be influencing predator habitat use and movement. Consistent with our hypothesis, dingoes and feral cats demonstrated significantly higher detection rates on game trails when compared to undisturbed vegetation at our study site. Dingoes exhibited the highest detection rates on game trails, with 97% of all detections occurring on trails. There was a similar pattern for cats, with 86% of detections occurring on game trails. Our results align with studies demonstrating the preference of these predators for using anthropogenic linear features (Dawson et al. 2018; Doherty et al. 2014; Raiter et al. 2018; Wysong et al. 2020). We suggest that the creation of game trails by megaherbivores could be an important, though overlooked, form of ecosystem engineering, with potentially far‐reaching ecological consequences for both predator and prey populations. While our findings can be used to refine both monitoring and management of these predators, key knowledge gaps remain regarding the broader ecological implications of this work.

The preferential use of game trails by dingoes and feral cats demonstrated by our study strongly aligns with literature from across Europe, North America, and Africa that has shown the propensity of terrestrial predators to use anthropogenic linear features, including the grey wolf (a congener, and ancestor, of the Australian dingo), African wild dog ( *Lycaon pictus* ) and red fox (Abrahms et al. 2016; Bischof et al. 2019; Dickie et al. 2017; Latham et al. 2011). Such a preference for linear features is often thought to reflect optimal foraging theory, whereby a predator adjusts its behaviour to maximise energy gain and/or minimise competition and mortality risk (MacArthur and Pianka 1966; Pyke et al. 1977). We speculate that game trails in tropical savannas may offer similar benefits to terrestrial predators as do other linear features (e.g., roads, seismic lines).

Linear features can influence predator ecology through multiple mechanisms. First, they can enhance movement efficiency by providing unobstructed travel corridors, allowing territorial predators to move faster and with less energetic cost. For example, in boreal forests, grey wolves and black bears ( *Ursus americanus* ) travel faster and cover longer distances along anthropogenic linear features compared to natural terrain (Dickie et al. 2017, 2020). For highly mobile and territorial species such as dingoes and feral cats (Edwards et al. 2001; Newsome et al. 2013; Wysong et al. 2020), this increased efficiency may enable more effective territory patrol, greater home range coverage, and potentially improved resource monopolisation. Second, linear features may improve visual and olfactory detection of prey by opening up dense vegetation, thereby enhancing encounter rates. In North America, seismic lines penetrating peatland habitats used by calving woodland caribou increased spatial overlap with and predation risk from wolves (DeMars and Boutin 2018). Similarly, in northern Australian savannas, game trails created by exotic megaherbivores often cut through dense vegetation as they connect patches of water, shade, and forage. These same trails may increase visibility for predators, especially in structurally complex habitats like riparian zones. When linear features penetrate key habitat refuges for prey species, they may reduce the protective value of these areas. In northern Australia's tropical savannas, riparian zones and floodplains are critical refuges for small native mammals such as the dusky rat ( *Rattus colletti* ) and pale field‐rat ( *Rattus tunneyi* ), which can occur at high densities in these habitats (Braithwaite and Griffiths 1996; Friend et al. 1988). Both species are preyed upon by dingoes (Corbett 1989) and feral cats (Leahy et al. 2016; Tuft et al. 2021). By providing direct predator access into these refuges, game trails may increase predation pressure and potentially create ecological traps or induce functional habitat loss, whereby prey either suffer higher mortality or avoid otherwise suitable habitat.

Game trails may also increase the hunting efficiency of feral cats. Cats typically hunt along habitat edges (Doherty et al. 2014) using an “ambush, stalk and pounce” strategy and locate prey using primarily visual and auditory cues (Moseby and McGregor 2022; Woinarski, Legge, and Dickman 2019). By creating a network of edges, game trails could increase feral cat hunting success in tropical savannas. Game trails also provide a clear hunting ground and adjacent thicker vegetation to hide behind, potentially improving their ability to detect and stalk prey. Feral cats preferentially target recently burnt firescars in northern Australian savannas (McGregor et al. 2016), most likely due to the increased predation efficacy following the removal of ground‐layer vegetation (Leahy et al. 2016; McGregor et al. 2015). However, in contrast to fire that generally occurs between May and October, game trails are maintained throughout the wet season, and may therefore be a key landscape feature for feral cats at this time of year. Given dingoes are coursing predators, game trails are less likely to provide a direct hunting gain but could still provide overall movement efficiencies or pathways to better hunting grounds (as suggested above). In Australia, direct evidence for predator dietary shifts associated with increasing densities of linear features is lacking. However, dingo diet was not affected by a dramatic increase in anthropogenic linear features (seismic testing lines) on active pastoral stations (i.e., an already modified landscape) in Western Australia (Duncan et al. 2022).

**FIGURE 4:**
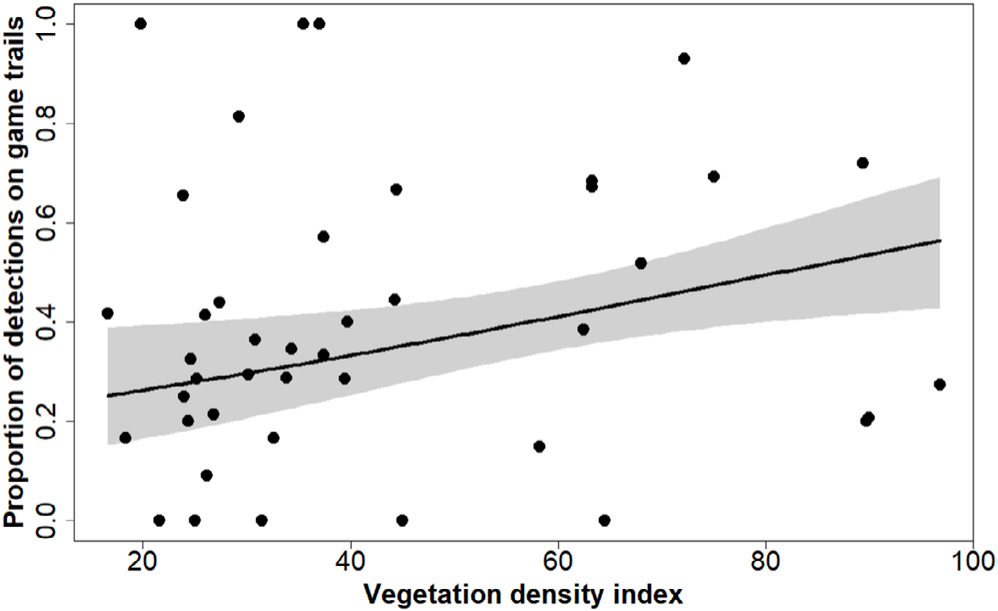
Modelled relationship between the proportion of northern brushtail possum detections on game trails and the density of the adjacent vegetation. Grey shading indicates the 95% confidence interval.

We also investigated the use of game trails by native mammals at risk of dingo and cat predation (Corbett 1989; Stokeld et al. 2018). The black‐footed tree‐rat was the only native mammal to exhibit a preference for game trails, and this preference was not influenced by ground‐layer vegetation density at a site. Game trails may therefore increase the exposure of this species to predation by dingoes and feral cats. The black‐footed tree‐rat is listed as Vulnerable under Australian legislation, and is known to be in significant decline on Melville Island (Davies et al. 2018; Neave et al. 2024). While the northern brushtail possum, also listed as Vulnerable under Australian legislation, tended to avoid game trails, this effect became muted as understorey density increased, such that in the densest vegetation there was no aversion to game trails (Figure 4). This may reflect a shift in the risk–reward trade‐off in dense vegetation, where the ability to move more quietly and efficiently along a game trail becomes more valuable to prey than avoiding potential exposure to predators.

Agile wallabies are a widespread and common species across the tropical savannas of Australia, and in our study area, they are the only native herbivore exceeding 15 kg in body mass (females ~15 kg, males up to 27 kg). They were also the most frequently detected species in our survey, yet exhibited a clear tendency to avoid game trails, with 62% of detections occurring on off‐trail cameras. This finding highlights a key distinction between the trail‐use behavior of native and exotic herbivores and suggests that trails formed by wallabies may not exert the same ecological influence—particularly with respect to shaping predator movement. Indeed, on neighboring Bathurst Island, where agile wallabies are present but exotic megaherbivores are absent, much of the landscape lacks any obvious network of game trails in comparison to Melville Island (pers. obs.).

We speculate that, by creating game trails, exotic megaherbivores could be supporting populations of dingoes and cats in Australian tropical savannas. Previously, the role of exotic megaherbivores in the decline of native mammals across northern Australian savannas has mostly focused on: (1) short‐term post‐fire grazing impeding ground‐layer vegetation recovery; and (2) longer‐term degradation of vegetation structure, both of which deplete important food and shelter resources, while exacerbating predation impacts (Legge et al. 2011, 2019; McGregor et al. 2014). Our concept of “micro‐corridors” caused by exotic megaherbivores represents a plausible new hypothesis that may further explain the broad association between predators, exotic megaherbivores, and native mammal decline across northern Australia (Davies et al. 2020; Stobo‐Wilson et al. 2020). Our study provides an important first step in demonstrating the preference of dingoes and cats for game trails; however, further research is required to understand the reasons for this preference, the spatial scales at which it operates, and whether these predators derive a fitness benefit from using game trails. Addressing these knowledge gaps will require high‐resolution movement data, diet analyses, coupled with robust predator and prey population monitoring. Without such data, we cannot ascertain to what degree predators benefit from game trails. For instance, it is possible that using game trails has no benefit to overall fitness, or that the benefits are minor and do not scale up to the population‐level. Moreover, the heightened detection of predators on trails may have resulted from dingoes and cats randomly encountering trails, and the trails funnelling their movement past camera traps, increasing detections rather than predators predominantly using trails. To address these uncertainties, future research should consider deploying multiple cameras along a single trail at varying distances, combined with high‐frequency GPS telemetry studies.

While both predator species were detected across our study sites, our design did not allow robust inferences about dingo‐cat interactions, such as possible behavioral avoidance or spatial partitioning. We acknowledge this as a limitation of our study but consider it important to briefly note the growing body of literature and interest in such interactions in Australia (Allen et al. 2015). This remains a potentially insightful avenue of research for future larger datasets from our study area.

Our study also has important implications for the monitoring and management of savanna predators, particularly feral cats. A significant challenge in landscape‐scale feral cat monitoring in northern Australia is the regionally low detectability of cats (Stokeld et al. 2018), necessitating considerable effort to achieve adequate detections for robustly modeling feral cat occurrence and density. This has also limited a broader understanding of the effectiveness of various ecosystem management strategies, such as early dry season burning and exotic megaherbivore control, in altering feral cat spatial occupancy and density in some locations. Our results suggest that predator surveys could be greatly enhanced by placing camera traps along game trails (Figure S1). The placement of cameras on other linear features, namely roads, has increased the detectability of cryptic invasive predators and has been demonstrated elsewhere in Australia (Geyle et al. 2020; Wysong, Lacona, et al. 2020). Game trails could also be targeted for cat control devices, such as Felixer Grooming Traps (Thylation, Adelaide, Australia; https://thylation.com). However, caution is warranted regarding the use of other less selective methods, such as poison baits on game trails, given that dingoes, considered ecologically and culturally important apex predators in savanna systems, are also frequently using these trails. Given that game trails are temporary linear features (unlike permanent roads), the removal of exotic megaherbivores could, over time, result in the gradual disappearance of these trails as vegetation recovers in their absence. This would also reduce any potential benefits that these trails provide to predators, with possible flow‐on effects for prey.

## 5 Conclusion

Our study is the first to explore how terrestrial predators use game trails created by exotic megaherbivores in Australia. We speculate that by creating a network of micro‐corridors, these megaherbivores may be exacerbating the impact of terrestrial predators on northern Australia's native fauna. Importantly, native fauna are simultaneously threatened by exotic megaherbivores and predators across virtually all Australian habitats. Yet, despite the potential continent‐wide impacts of game trails, their impact remains poorly understood.

## Author Contributions

**Georgina Neave:** conceptualization (equal), data curation (lead), formal analysis (equal), investigation (lead), methodology (equal), writing – original draft (lead), writing – review and editing (equal). **Brett P. Murphy:** conceptualization (equal), funding acquisition (lead), methodology (equal), project administration (lead), resources (lead), supervision (equal), writing – review and editing (equal). **Tiwi Rangers:** conceptualization (equal), investigation (equal). **Hugh F. Davies:** conceptualization (lead), formal analysis (equal), investigation (supporting), methodology (equal), supervision (equal), writing – review and editing (equal).

## Ethics Statement

This research was conducted under Charles Darwin University Animal Ethics Committee Permit A22006 and the Northern Territory Parks and Wildlife Commission Scientific Research Permit 69229. This research was undertaken as part of a research agreement between Charles Darwin University and the Tiwi Land Council. The Tiwi Islands are Aboriginal freehold land owned by the Tiwi Traditional Owners. Tiwi Land Council serves as their representative body, as mandated under the Aboriginal Land Rights (Northern Territory) Act (1976).

## Conflicts of Interest

The authors declare no conflicts of interest.

## Supporting information


**Appendix S1.** Supporting Information.
Figure S1.

## Data Availability

Data available from the Dryad Digital Repository https://doi.org/10.5061/dryad.0zpc86776.
