# Supplementary material for: Exotic Megaherbivores as Ecosystem Engineers in Australian Savannas: Do They Facilitate Predator Movement?
Source: Ecol Evol. 2025 Jul 9;15(7):e71622. doi: 10.1002/ece3.71622 (PMC12240593; doi:10.1002/ece3.71622)
Supplement: Supplementary file 1 — Appendix S1. Supporting Information. Figure S1. [file ECE3-15-e71622-s001.docx]

**SUPPLEMENTARY INFORMATION**

Exotic Megaherbivores as Ecosystem Engineers in Australian Savannas: Do They Facilitate Predator Movement?

Georgina Neave | Brett P. Murphy | Tiwi Rangers | Hugh F. Davies

Correspondence:

Georgina Neave (georgina.neave@proton.me)

**Table S1.** Description and justification of the explanatory variables used in analyses to investigate biotic interactions that may influence a species’ use of game trails on Melville Island.

| **Explanatory variable** | **Description and justification for inclusion in the analysis** | **Species modelled** |
| --- | --- | --- |
| Vegetation density | The average height at which a pole could no longer be seen through vegetation (see methods). Used to account for differences in vegetation structure between sites. Predators are more likely to use habitats where their preferred prey are more abundant and/or easier to capture. Game trails could afford more benefits to predators in dense vegetation. | - Cat - Dingo - Black-footed tree-rat - Northern brown bandicoot - Northern brushtail possum - Agile wallaby |
| Dingo activity | The number of independent (> 30 min apart) dingo detections recorded at each site’s on-trail camera. Used to investigate the potential negative influence of dingoes on cats (Brook et al., 2012; Glen & Dickman, 2005; Kennedy et al., 2012) and the various species predated on (Corbett, 2001; Stokeld et al., 2018). | - Cat - Black-footed tree-rat - Northern brown bandicoot - Northern brushtail possum - Agile wallaby |
| Cat activity | The number of independent (> 30 min apart) cat detections recorded at each site’s on-trail camera. Used to investigate the influence of cat presence on prey species (Kutt, 2012; Murphy et al., 2019; Stokeld et al., 2018) and potential interspecific interactions with dingoes. | - Dingo - Black-footed tree-rat - Northern brown bandicoot - Northern brushtail possum - Agile wallaby |
| Exotic megaherbivore activity | The number of independent (> 30 min apart) exotic megaherbivore (buffalo and horse) detections recorded at each site’s on-trail camera. Used as an indicator of trail definition in the landscape to determine if trail use by certain species is more likely when trails are maintained by high megaherbivore traffic. | - Cat - Dingo - Black-footed tree-rat - Northern brown bandicoot - Northern brushtail possum - Agile wallaby |
| Native mammalian prey activity | The total number of independent detections (> 30 min apart) of all species of native mammalian prey (10−2500 g) at each site’s on-trail camera. Includes ‘critical weight range’ species which are a size group of native mammal species considered most at risk of extinction from predation by introduced predators (Murphy & Davies, 2014). Used as an indicator of prey availability to cats and dingoes. | - Cat - Dingo |
| Agile wallaby activity | The number of independent (> 30 min apart) agile wallaby detections recorded at each site’s on-trail camera. Used in analyses as an indicator of prey availability at each site for dingoes. Agile wallaby are a significant prey item recorded in dingo diet analyses (Corbett, 2001; Duncan et al., 2022; Morrant et al., 2017; Stokeld et al., 2018). | - Dingo |

**Table S1 References:**

Brook, L. A., Johnson, C. N., & Ritchie, E. G. (2012). Effects of predator control on behaviour of an apex predator and indirect consequences for mesopredator suppression. *Journal of Applied Ecology*, *49*(6), 1278–1286. https://doi.org/10.1111/j.1365-2664.2012.02207.x

Corbett, L. K. (2001). *The dingo in Australia and Asia*. J. B. Books.

Duncan, T. A., Fleming, P. A., & Dawson, S. J. (2022). Diet of dingoes in the West Kimberley, and the impact of linear clearing. *Australian Mammalogy*, *44*(3), 338–346. https://doi.org/10.1071/AM21016

Glen, A. S., & Dickman, C. R. (2005). Complex interactions among mammalian carnivores in Australia, and their implications for wildlife management. *Biological Reviews*, *80*(03), 387. https://doi.org/10.1017/S1464793105006718

Kennedy, M., Phillips, B. L., Legge, S., Murphy, S. A., & Faulkner, R. A. (2012). Do dingoes suppress the activity of feral cats in northern Australia?: Dingoes suppress cats in north Australia. *Austral Ecology*, *37*(1), 134–139. https://doi.org/10.1111/j.1442-9993.2011.02256.x

Kutt, A. S. (2012). Feral cat (*Felis catus*) prey size and selectivity in north-eastern Australia: Implications for mammal conservation. *Journal of Zoology*, *287*(4), 292–300. https://doi.org/10.1111/j.1469-7998.2012.00915.x

Morrant, D. S., Wurster, C. M., Johnson, C. N., Butler, J. R. A., & Congdon, B. C. (2017). Prey use by dingoes in a contested landscape: Ecosystem service provider or biodiversity threat? *Ecology and Evolution*, *7*(21), 8927–8935. https://doi.org/10.1002/ece3.3345

Murphy, B. P., & Davies, H. F. (2014). There is a critical weight range for Australia’s declining tropical mammals: Critical weight range for Australia’s declining tropical mammals. *Global Ecology and Biogeography*, *23*(9), 1058–1061. https://doi.org/10.1111/geb.12173

Murphy, B. P., Woolley, L.-A., Geyle, H. M., Legge, S. M., Palmer, R., Dickman, C. R., Augusteyn, J., Brown, S. C., Comer, S., Doherty, T. S., Eager, C., Edwards, G., Fordham, D. A., Harley, D., McDonald, P. J., McGregor, H., Moseby, K. E., Myers, C., Read, J., … Woinarski, J. C. Z. (2019). Introduced cats (*Felis catus*) eating a continental fauna: The number of mammals killed in Australia. *Biological Conservation*, *237*, 28–40. https://doi.org/10.1016/j.biocon.2019.06.013

Stokeld, D., Fisher, A., Gentles, T., Hill, B., Triggs, B., Woinarski, J. C. Z., & Gillespie, G. R. (2018). What do predator diets tell us about mammal declines in Kakadu National Park? *Wildlife Research*, *45*(1), 92–101. https://doi.org/10.1071/WR17101

**Table S2.** Summary of naïve occupancy, total number of independent detections (>30 min apart), and mean detections per camera on and off game trails across the full survey period for all study taxa.

| **Species** | **Naïve site occupancy (%)** | **Total number of independent detections** | **Mean number of detections off-trail (±SE)** | **Mean number of detections on-trail (±SE)** |
| --- | --- | --- | --- | --- |
| **Dingo**  (*Canis familiaris*) | 76 | 386 | 0.28 (0.08) | 9.61 (2.73) |
| **Cat**  (*Felis catus*) | 51 | 82 | 0.42 (0.13) | 2.73 (0.55) |
| **Black-footed tree-rat** (*Mesembriomys gouldii melvillensis*) | 35 | 60 | 1.11 (0.30) | 2.22 (0.62) |
| **Northern brown bandicoot** (*Isoodon macrourus*) | 69 | 332 | 4.4 (0.91) | 5.09 (0.90) |
| **Northern brushtail possum**  (*Trichosurus vulpecula arnhemensis*) | 82 | 940 | 13.14 (1.90) | 9.17 (1.80) |
| **Agile wallaby**  (*Macropus agilis*) | 84 | 2045 | 29.51 (3.92) | 18.05 (2.84) |
| **Horse**  (*Equus caballus*) | 67 | 205 | 1.21 (0.25) | 4.82 (0.92) |
| **Buffalo**  (*Bubalus bubalis*) | 98 | 482 | 0.98 (0.23) | 8.64 (1.10) |
| **Small mammals** (*Pseudomys*, *Rattus* and *Sminthopsis* spp.) | 16 | 25 | 1.25 (0.73) | 1.88 (0.67) |

**Table S3.** Model ranking table for generalised linear models of species' preference for game trails, as a function of biotic variables. Only models with ΔQAIC < 2 are shown. + indicates variables used in each model. Grey shading indicates statistically significant variables. NA used for variables not included in species’ models. ΔQAIC is Delta Akaike Information Criterion for Quasi-Likelihood, w_i_ is the Akaike weight.

| **Species** | **Vegetation density** | **Dingo activity** | **Cat activity** | **Exotic mega-herbivore activity** | **Native prey activity** | **Agile wallaby activity** | **ΔQAIC** | **w_i_** |
| --- | --- | --- | --- | --- | --- | --- | --- | --- |
| **Dingo**  (*Canis familiaris*) |  | NA | + |  |  |  | 0.00 | 0.17 |
|  |  | NA | + |  |  | + | 0.99 | 0.10 |
|  |  | NA |  |  |  |  | 1.44 | 0.08 |
|  |  | NA | + |  | + |  | 1.60 | 0.08 |
|  |  | NA | + | + |  |  | 1.79 | 0.07 |
| **Cat**  (*Felis catus*) |  |  | NA |  |  | NA | 0.00 | 0.25 |
|  |  |  | NA | + |  | NA | 0.17 | 0.22 |
|  |  |  | NA | + | + | NA | 1.94 | 0.09 |
| **Black-footed tree-rat**  (*Mesembriomys gouldii melvillensis*) |  |  |  |  | NA | NA | 0.00 | 0.39 |
| **Northern brown bandicoot**  (*Isoodon macrourus*) |  |  |  |  | NA | NA | 0.00 | 0.29 |
|  |  |  | + |  | NA | NA | 1.30 | 0.15 |
|  |  | + |  |  | NA | NA | 1.88 | 0.11 |
| **Northern brushtail possum**  (*Trichosurus vulpecula arnhemensis*) | + |  |  |  | NA | NA | 0.00 | 0.20 |
|  | + |  |  | + | NA | NA | 0.75 | 0.14 |
|  | + |  | + | + | NA | NA | 0.99 | 0.12 |
|  | + |  | + |  | NA | NA | 1.00 | 0.12 |
|  | + | + |  |  | NA | NA | 1.75 | 0.08 |
| **Agile wallaby** (*Macropus agilis*) |  |  |  | + | NA | NA | 0.00 | 0.14 |
|  | + |  |  | + | NA | NA | 0.06 | 0.13 |
|  |  | + |  | + | NA | NA | 0.87 | 0.09 |
|  |  |  | + |  | NA | NA | 0.90 | 0.09 |
|  |  |  |  |  | NA | NA | 1.32 | 0.07 |
|  |  |  | + | + | NA | NA | 1.51 | 0.06 |
|  | + |  |  |  | NA | NA | 1.52 | 0.06 |
|  |  | + | + |  | NA | NA | 1.70 | 0.06 |
|  | + | + |  | + | NA | NA | 1.70 | 0.06 |

**Figure S1.** Cumulative nightly detection probabilities for dingoes (*Canis familiaris*) and feral cats (*Felis catus*) are shown for cameras placed on game trails (blue line; blue shading = 95% confidence intervals) versus in undisturbed vegetation (red line; red shading = 95% confidence intervals). To examine the effect of camera placement (on-trail vs. off-trail) on nightly detection probability, we used Bayesian generalised linear mixed models (GLMMs). Models were fitted using the blme package in R version 4.3.1 (R Core Team, 2023). Each species was modelled separately, with the response variable specified as the number of nights detected versus not detected (binomial distribution). Camera placement was included as a fixed effect, and 'site' was included as a random effect to account for non-independence among cameras at the same location. We generated cumulative nightly detectability curves for each species, truncated at 200 nights following Moore et al. (2020).


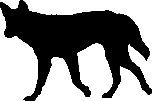

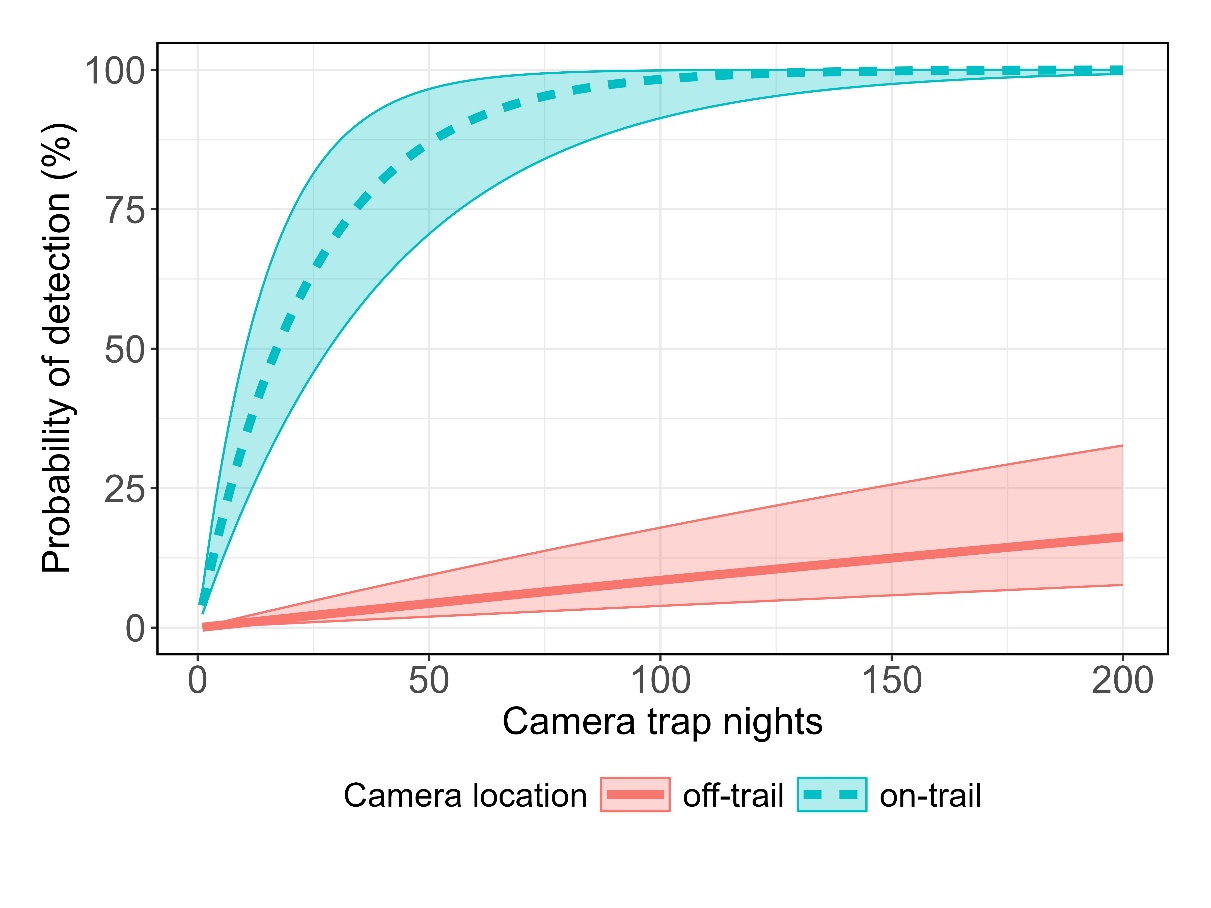


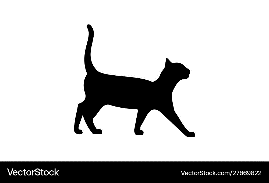

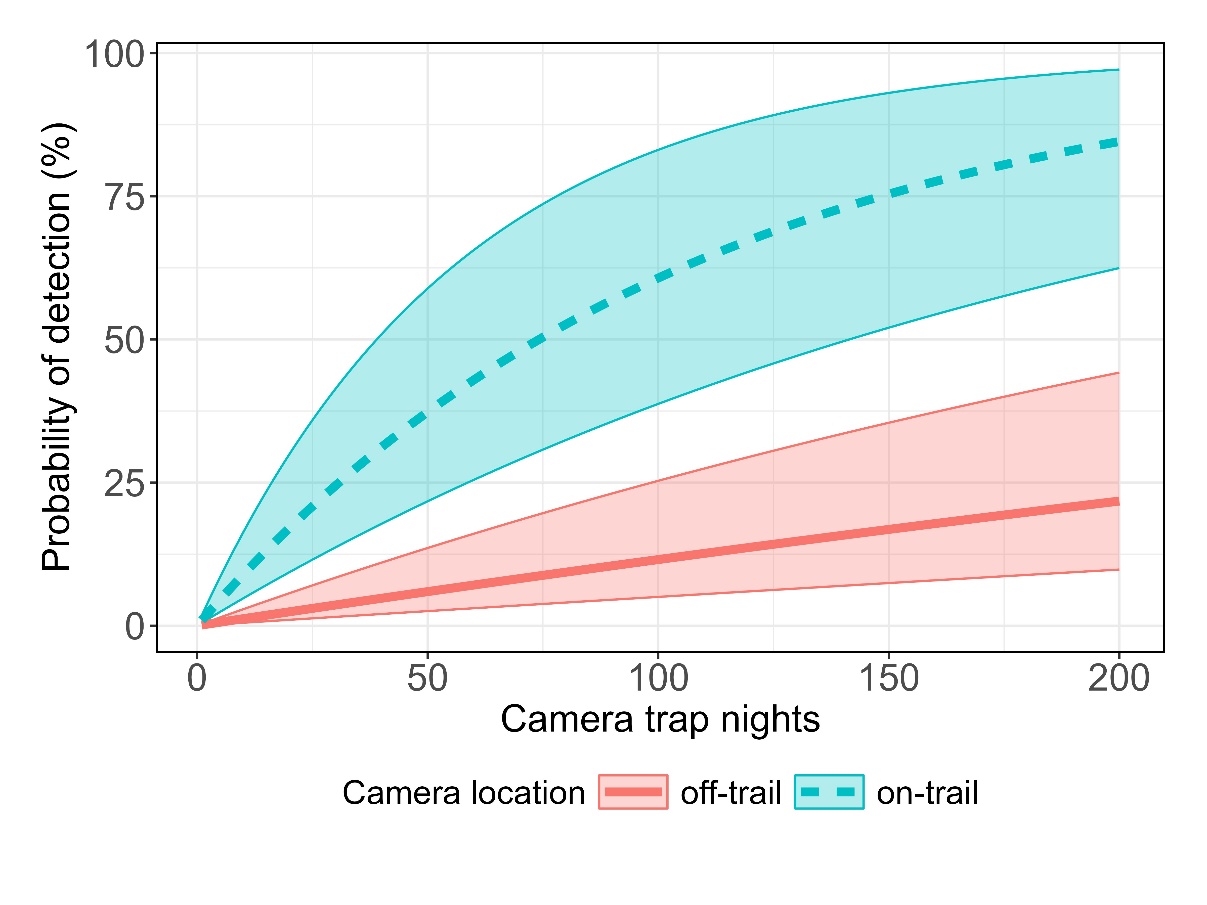


References for Figure S1:

Moore, H. A., Valentine, L. E., Dunlop, J. A., & Nimmo, D. G. (2020). The effect of camera orientation on the detectability of wildlife: A case study from north-western Australia. *Remote Sensing in Ecology and Conservation*, *6*(4), 546–556. https://doi.org/10.1002/rse2.158
